# Association between Temperature and Emergency Room Visits for Cardiorespiratory Diseases, Metabolic Syndrome-Related Diseases, and Accidents in Metropolitan Taipei

**DOI:** 10.1371/journal.pone.0099599

**Published:** 2014-06-16

**Authors:** Yu-Chun Wang, Yu-Kai Lin

**Affiliations:** 1 Department of Bioenvironmental Engineering, College of Engineering, Chung Yuan Christian University, Jungli City, Taiwan; 2 Research Center for Environmental Risk Management, Chung Yuan Christian University, Jungli City, Taiwan; 3 Environmental and Occupational Medicine and Epidemiology Program, Department of Environmental Health, Harvard School of Public Health, Boston, Massachusetts, United States of America; 4 Institute of Environmental Health, College of Public Health, National Taiwan University, Taipei, Taiwan; The Ohio State University, United States of America

## Abstract

**Objective:**

This study evaluated risks of the emergency room visits (ERV) for cerebrovascular diseases, heart diseases, ischemic heart disease, hypertensive diseases, chronic renal failure (CRF), diabetes mellitus (DM), asthma, chronic airway obstruction not elsewhere classified (CAO), and accidents associated with the ambient temperature from 2000 to 2009 in metropolitan Taipei.

**Methods:**

The distributed lag non-linear model was used to estimate the cumulative relative risk (RR) and confidence interval (CI) of cause-specific ERV associated with daily temperature from lag 0 to lag 3 after controlling for potential confounders.

**Results:**

This study identified that temperatures related to the lowest risk of ERV was 26 °C for cerebrovascular diseases, 18 °C for CRF, DM, and accidents, and 30 °C for hypertensive diseases, asthma, and CAO. These temperatures were used as the reference temperatures to measure RR for the corresponding diseases. A low temperature (14°C) increased the ERV risk for cerebrovascular diseases, hypertensive diseases, and asthma, with respective cumulative 4-day RRs of 1.56 (95% CI: 1.23, 1.97), 1.78 (95% CI: 1.37, 2.34), and 2.93 (95% CI: 1.26, 6.79). The effects were greater on, or after, lag one. At 32°C, the cumulative 4-day RR for ERV was significant for CRF (RR = 2.36; 95% CI: 1.33, 4.19) and accidents (RR = 1.23; 95% CI: 1.14, 1.33) and the highest RR was seen on lag 0 for CRF (RR = 1.69; 95% CI: 1.01, 3.58), DM (RR = 1.69; 95% CI: 1.09, 2.61), and accidents (RR = 1.19; 95% CI: 1.11, 1.27).

**Conclusions:**

Higher temperatures are associated with the increased ERV risks for CRF, DM, and accidents and lower temperatures with the increased ERV risks for cerebrovascular diseases, hypertensive diseases, and asthma in the subtropical metropolitan.

## Introduction

Extreme temperature on a given day and prolonged extreme heat and cold events (referred to in this paper simply as “events”) are associated worldwide with increased mortality from, and morbidity of, all causes or cardiovascular diseases, respiratory diseases, and renal diseases [1]–[13]. Only a few studies have addressed the effect of extreme temperatures on specific diseases [1], [9], [12]–[14]. These findings result in better general public health preparedness for extreme heat and cold events. However, to raise awareness of adverse effects and reduce the additional medical expenditure associated with extreme temperatures, it is of interest to examine associations between acute measures of highly susceptible subpopulations and the effect of extreme temperatures. Information on temperature-health relationships and threshold temperatures for specific diseases is important in establishing a timely extreme temperature health warning system.

Metropolitan Taipei (Taipei City and New Taipei City), located at 25°N and 121°E, has a hot and humid climate with daily mean temperature ranges from 8 °C in winter to 33 °C in summer. The population size is about 6.41 million, of which 9.74% are the elderly. How extreme temperatures may exacerbate disease for a population living in this subtropical climate is of interest. In this study, an extreme temperature event was defined as a daily average temperature in the 97^th^ or 5^th^ percentile lasting for 3 days or longer or temperatures in the 99^th^ or 1^st^ percentile lasting for 2 days or longer.

The aim of this ecological study was to evaluate the associations between ambient temperature, extreme temperature events, and the first extreme heat or cold event of the year and risks for emergency room visits (ERV) for cerebrovascular diseases, heart diseases, ischemic heart disease (IHD), hypertensive diseases, chronic renal failure (CRF), diabetes mellitus (DM), asthma, chronic airway obstruction not elsewhere classified (CAO), and accidents. This is the first study to analyze the relationship between cause-specific ERV and temperature in subtropical areas, using the distributed lag non-linear model (DLNM) to evaluate the nonlinear association and cumulative risks related to ambient temperature at various lag days.

## Materials and Methods

### Data source

The present study used daily meteorological records from the Central Weather Bureau, universal health insurance claims data from the National Health Research Institute, and daily air pollution monitoring records from the Taiwan Environmental Protection Administration for metropolitan Taipei from 2000 to 2009.

Since 1996, over 96% of the 23 million population of Taiwan have been covered by the Taiwan National Health Insurance program [15]. Using the electronic reimbursement claim records, the Taiwan National Health Research Institute established a cohort for research purposes consisting of a nationally representative population of one million people randomly sampled from all insured residents [16]. In 2000, approximately 31.8% of these one million people resided in the metropolitan Taipei. The dataset contained scrambled identification numbers of the citizens and information on gender, birth date, health care received, physicians' diagnoses at outpatient visits, inpatient admissions and discharges, use of emergency services, and the medical care providers involved. Disease diagnoses were coded according to the 9th revision of the International Classification of Diseases with Clinical Modification (ICD9 CM). The records of ERV for cerebrovascular diseases (ICD9 CM 430–438), heart diseases (ICD9 CM 391, 402, 404, 415, 416, 785, 393–398, and 420–429), IHD (ICD9 CM 410–414), hypertensive diseases (ICD9 CM 401–404), CRF (ICD9 CM 585), DM, (ICD9 CM 250), asthma (ICD9 CM 493), CAO (ICD9 CM 496), and accidents (ICD9 CM 800-999) during the study period were retrieved.

The Taiwan Air Quality Monitoring Network established by the Taiwan Environmental Protection Administration in 1993 included 74 stationary monitoring stations distributed throughout the island [17]. Concentrations of ambient air pollutants, such as particulate matters less than 10 µm in aerodynamic diameter (PM_10_), nitrogen oxides (NOx), and ozone (O_3_), were determined and recorded hourly at each station. The present study analyzed the daily average data for PM_10_, O_3_, and NOx monitored at 13 general ambient stations in Taipei over the study period.

The Central Weather Bureau provided 24-hour weather data (average temperature, maximum temperature, minimum temperature, relative humidity, wind speed, and barometric pressure) from 25 real-time weather monitoring stations in Taiwan [18]. The present study used daily weather measurements from the Taipei weather station collected from 2000 to 2009 [18]. Please refer the previous report [19] to see the locations for 13 general ambient stations and Taipei weather station.

### Definition of extreme temperature events

To assess the additional effects of prolonged extreme temperature (“events”), we created a variable *Extreme* which described the daily ambient temperature of the study period as normal temperature or extreme heat or cold. Previous reports have addressed detailed methods for assessing the effect of extreme events [10], [11]. In brief, we evaluated the ERV risks associated with 10 types of extreme temperature event consisting of temperatures in the 97^th^ (n = 114 days) or 5^th^ (n = 187 days) percentile lasting for 3–5, 6–8, or >8 days or temperatures in the 99^th^ (n = 37 days) or 1^st^ (n = 41 days) percentile lasting for 2–3 days or >3 days. All days without any event were classed as having normal temperatures. In addition, the first extreme heat or cold event of the year (97^th^ or 5^th^ temperature percentile, respectively) lasting 3 days or longer was coded as an extra categorical dummy variable (*First*) to assess adverse effects for populations exposed to the first extreme temperature event of the year [10].

### Non-linear association between daily temperature and cause-specific ERV, and the relative risk for extreme temperature events

The association between the daily average temperatures and daily cause-specific ERV was evaluated using a distributed lag non-linear model (DLNM) with Poisson distribution. Natural cubic spline (NS) DLNM models were used to analyze the non-linear and delayed effects of temperature and air pollutants. Relative risks (RR) due to temperature and air pollutants were estimated using the cross-basis function in DLNM models, as described previously [20], [21]. The cross-basis function contains the dimensions of variables and lag days. This study placed the knots of variables at equally spaced quantiles of the predictor, and the knots of lag at equally spaced values on the log scale of lags.

The covariate “daily average temperature” was set at NS with 5 degrees of freedom (*df*). To estimate the acute effects of ambient temperature, the cumulative 4-day (lag 0 to lag 3 [3], lag set at 3 *df*) RR and 95% confidence interval (CI) of cause-specific ERV were estimated by comparing the risk associated with the extreme temperatures of 14 °C and 32 °C to that at the temperature resulting in the lowest cause-specific ERV (the centered temperature). In Taipei, the 5th percentile of the average temperature was about 14 °C and the 99th percentile was about 32 °C during 2000-2009.

The defined extreme temperature events (*Extremes*) and the first extreme temperature events of the year (*First*) were set as categorical covariates and risks associated with these events estimated by comparison with risks on days defined as normal temperature (nonconsecutive days of extreme temperature and days of normal temperature).

Concentrations of air pollutants PM_10_, NOx, and O_3_ were set at NS with 5 *df*. Six-day cumulative effects (lag 0 to lag 5, 3 *df* for lag space) were estimated by comparing the concentrations of air pollutants at the 75th (Q3) percentile to the concentrations at the 25th (Q1) percentile [22], [23].

The model for the expected cause-specific ERV count on day (t) is
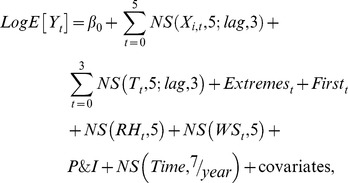
where 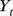 is the expected cause-specific ERV on day t, 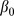 the model intercept, and 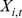 the NS for measurements of air pollutants (*i* = 1–3 for PM_10_, NOx, and O_3_, *df* = 5) on day t, and effects were accumulated for 6 days (lag 0 to lag 5) with the lag space set at 3 *df*. 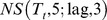 is the NS for the daily average temperature. The temperatures were set at 5 *df* and their effects accumulated for 4 days (lag 0 to lag 3) with the lag space set at 3 *df*. 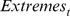 is the categorical variable representing extreme temperature events on day t., and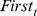 indicates the city-specific first extreme heat or cold event of the year. The NS function with 5 *df* was also used in the daily measurement of relative humidity (RH) and wind speed (WS). The smoothing time term (*Time*) was set at 7 *df* per year. Other covariates, such as holidays, day of the week, and daily ERV for pneumonia and influenza (*P&I*, ICD9 CM 480–487), were also adjusted in the models.

Sensitivity analysis was used to evaluate *df*, which ranged from 4 to 6 for the temperature-ERV curves. Time smoothing with *df*s of 4, 7, and 14 per year was also performed. Akaike's information criterion was used for model selection [24]. All data manipulation and statistical analyses were performed using SAS version 9.1 (SAS Institute Inc., Cary, NC, USA) and Statistical Environment R 2.15.

## Results

Table 1 lists the characteristics of the ambient environment and cause-specific ERV for metropolitan Taipei, from 2000 to 2009. Among 192089 insured persons, we identified 6962 ERV for cerebrovascular diseases, 5198 for heart diseases, 5743 for IHD, 7366 for hypertensive diseases, 2610 for CRF, 3573 for DM, 835 for asthma, 2371 for CAO, and 141792 for accidents. Of whom, 63.3%, 54.4%, 58.3%, 50.7%, 55.3%, 57.6%, 14.0%, 82.6%, and 10.9%, respectively, were the elderly.

**Table 1:** Characteristics of the ambient environment and causes for emergency room visits in metropolitan Taipei from 2000 to 2009.

	Mean	Standard deviation	Minimum	25^th^	50^th^	75^th^	Maximum
Ambient environment							
Average temperature, °C	23.4	5.28	8.30	19.3	23.9	28.0	33.0
Relative humidity, %	75.7	9.12	37.0	69.0	75.0	83.0	98.0
Wind speed, m/sec	2.61	1.22	0	1.60	2.40	3.60	7.90
PM_10_, µg/m^3^	48.4	23.6	10.7	31.4	43.6	60.1	286
NOx, ppb	33.0	14.4	3.90	23.1	30.1	39.5	119
O_3_, ppb	25.5	9.32	4.53	19.0	24.9	31.2	73.1
Emergency room visits (visits/day)							
Cerebrovascular diseases	2	1	0	1	2	3	9
Heart diseases	1	1	0	1	1	2	7
Ischemic heart disease	2	1	0	1	1	2	7
Hypertensive diseases	2	2	0	1	2	3	11
Chronic renal failure	1	1	0	0	0	1	5
Diabetes mellitus	1	1	0	0	1	2	6
Asthma	0	1	0	0	0	0	5
Chronic airway obstruction not elsewhere classified	1	1	0	0	0	1	6
Accidents	39	8	11	33	38	44	83
Pneumonia and influenza	2	1	0	1	1	3	11

### Cumulative 4-day temperature effects

**Figure 1:**
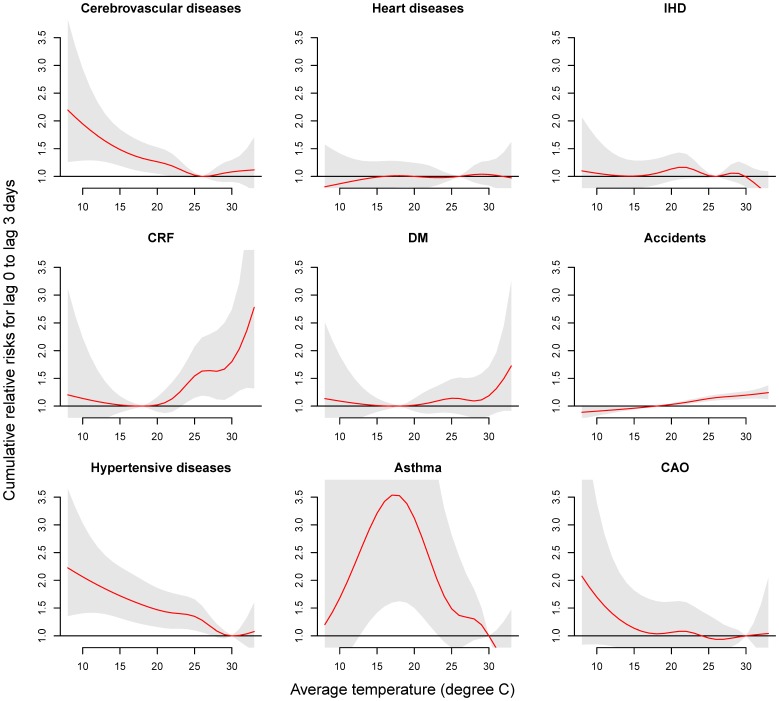
Associations between cause-specific emergency room visits and daily average temperatures in metropolitan Taipei from 2000 to 2009. Cumulative 4-day RRs were estimated using DLNM and a centered temperature of 26 °C for cerebrovascular diseases, 18 °C for CRF, DM, and accidents, and 30 °C for hypertensive diseases, asthma, and CAO.

The effects of average temperatures and extreme temperature events on ERV were estimated using DLNM after controlling for daily average levels of PM_10_, NOx, O_3_, RH, WS, daily ERV for pneumonia and influenza, holidays, day of the week, and long-term trends. Figure 1 shows the associations between the daily average temperature and cause-specific ERV. The lowest ERV were associated with an average temperature of 26 °C in the case of cerebrovascular diseases, 18 °C for CRF, DM, and accidents, and 30 °C for hypertensive diseases, asthma, and CAO; these temperatures were used as the centered temperatures for the corresponding diseases. At 14 °C (*i.e*. the 5th percentile temperature), the cumulative 4-day risk of ERV compared to that at the centered temperature was significant for cerebrovascular diseases with a RR of 1.56 (95% CI: 1.23, 1.97), hypertensive diseases with a RR of 1.78 (95% CI: 1.37, 2.34), and asthma with a RR of 2.93 (95% CI: 1.26, 6.79). In contrast, that for CRF or accidents was significantly associated with a temperature of 32 °C (*i.e*. 99th percentile temperature), with respective RRs of 2.36 (95% CI: 1.33, 4.19) and 1.23 (95% CI: 1.14, 1.33).

### Delayed effects of temperature

**Figure 2:**
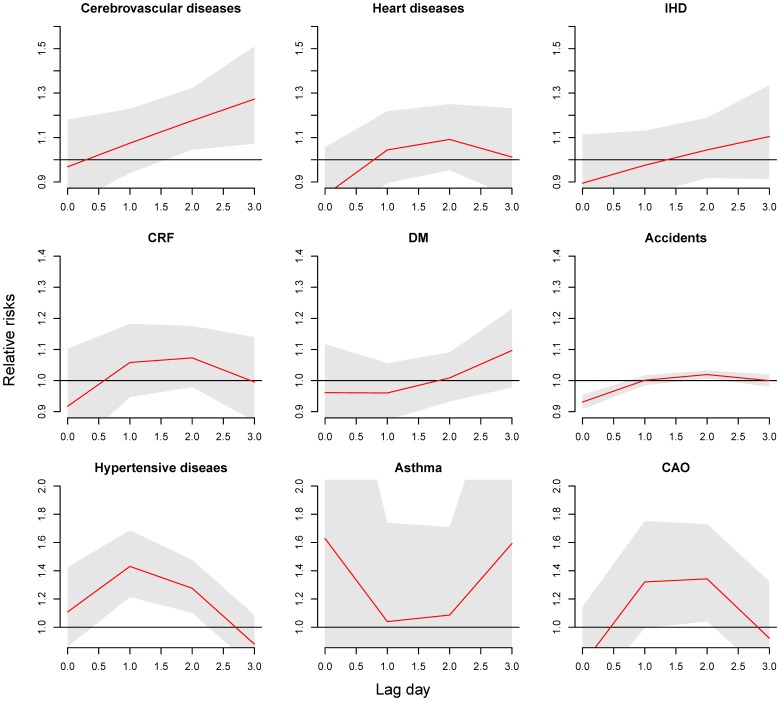
Lag effects of a temperature of 14°C on cause-specific emergency room visits compared to a centered temperature of 26 °C for cerebrovascular diseases, 18 °C for CRF, DM, and accidents, and 30 °C for hypertensive diseases, asthma, and CAO.

Figure 2 shows the cause-specific RRs in different lags at 14 °C, peaked on, or after lag 1. The RR was the highest at lag 3 for cerebrovascular diseases (RR = 1.27, 95% CI: 1.07, 1.51), lag 1 for hypertensive diseases (RR = 1.43; 95% CI: 1.21, 1.69), and lag 1-2 for CAO (RR = 1.34; 95% CI: 1.04, 1.73), while ERV for asthma were not associated with temperature on any lag day.

**Figure 3:**
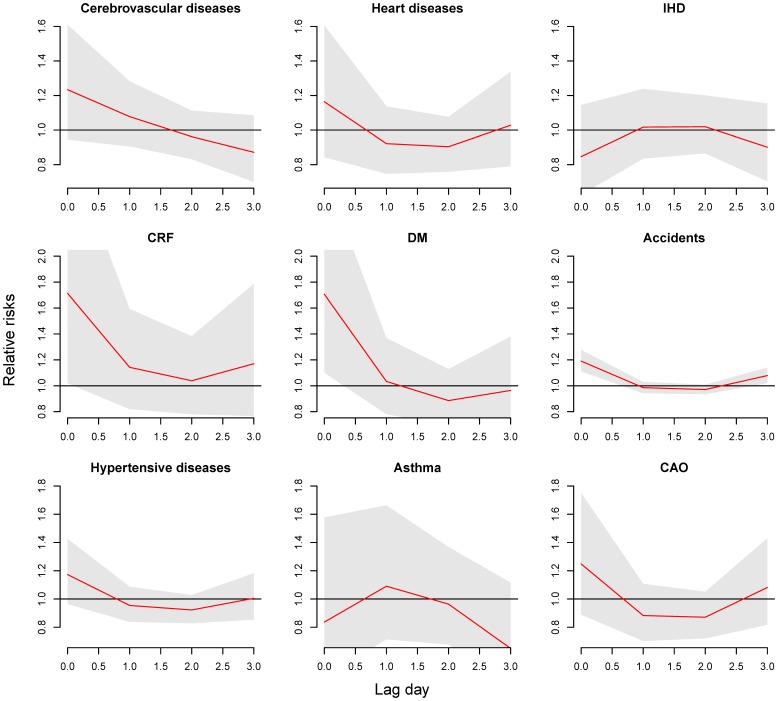
Lag effects of a temperature of 32°C on cause-specific emergency room visits compared to a centered temperature of 26 °C for cerebrovascular diseases, 18 °C for CRF, DM, and accidents, and 30 °C for hypertensive diseases, asthma, and CAO.

As shown in Figure 3, with the exception of IHD and asthma, an extreme high temperature of 32 °C resulted in the highest risk for cause-specific ERV on lag 0. The RR of ERV being 1.69 for CRF (95% CI: 1.01, 3.58), 1.69 for DM (95% CI: 1.09, 2.61), and 1.19 for accidents (95% CI: 1.11, 1.27). Although the temperature related ERV risk was also the highest on lag 0 for cerebrovascular diseases, heart diseases, hypertensive diseases, and CAO, the risks were not significant.

### Risks from prolonged extreme temperatures

This study did not identify any significant risks for extreme heat and cold events and their first occurrence in the year, except for ERV for asthma and CRF. The RR of ERV for asthma was 2.97 (95% CI: 1.00–8.81) when the study population was exposed to the 5^th^ percentile extreme temperature for 6–8 days (data not shown). In addition, the RR of ERV for CRF was 3.12 (95% CI: 1.09–8.92) when the population was exposed to the 1^st^ percentile extreme temperature for >3 days or longer (data not shown).

## Discussion

This is the first study, to the best of our knowledge, to evaluate the effect of daily temperature and extreme temperature events on the ERV risks for cardiorespiratory diseases and metabolic syndrome-related diseases in a subtropical metropolitan area. Based on findings in this study and previous studies [5], [10], [11], [25]–[27], the temperature-ERV associations are very different from temperature-mortality associations. Previous studies have shown that, in the case of cardiovascular diseases, high temperature is associated with marked and acute effects on mortality [11], [28], whereas morbidity is not significantly associated with the temperature [2]–[5]. In contrast, morbidity of respiratory diseases is associated with extreme heat [1]–[5], [29]. Our study found that ERV for cerebrovascular diseases, hypertensive diseases, and asthma were associated with low temperatures and those ERV for CRF, DM, and accidents were associated with high temperatures.

Exposure to high temperatures could increase plasma viscosity and cholesterol levels in serum, resulting in higher blood pressure [30]. In addition, thermoregulation of body is inhibited, blood shifting to underneath the skin surface to cool down the body temperature that further increases the pressure on heart and lung [31]. On the other hand, low temperatures could stress cardiovascular system due to the blood pressure fluctuation and hematological change, leading to cold-induced vasoconstriction and consequent loss of plasma fluid, predisposing the exposed subjects to arterial thrombosis [28]. Hong et al. have reported that cardiovascular markers, such as low-density lipoprotein cholesterol concentration may increase and high-density lipoprotein cholesterol level may decrease during exposure to decreasing ambient temperature. This mechanism may explain the excess cardiovascular mortality in cold weather [32].

Previous studies indicated that the wind draws heat away from the exposed body and makes exposed people feel cold [33]. The strong wind may enhance the cold effect [34]. Significant association between wind and mortality from and morbidity of cardiovascular diseases had been reported [35], [36]. In addition, the low wind may increase concentrations of air pollutants, leading to increased respiratory health effects [37]. This study thus estimated the associations between temperatures and cause-specific ERV after controlling for the potential effect of the wind speed.

Honda et al. have evaluated the optimum temperature with the lowest mortality, is around the 80-85th percentile values of daily maximum temperatures [38]. In the present study, the threshold temperatures of ERV for evaluated morbidities ranged from 18 °C to 30 °C. Several studies have reported threshold temperatures associated with cause-specific morbidity [1], [5], [39]. Lin et al. (2009) found that the threshold temperature ranging from 28.9 °C to 29.4 °C was critical for hospitalization of patients with respiratory and cardiovascular diseases in New York City [1]. Kovats et al. [5] reported that the threshold temperature varies with disease, ranging from 6 °C to 24 °C in Greater London. The threshold temperature for ERV and death in Brisbane is 27 °C [39]. Thus, it is unlikely that we can determine an optimum temperature for morbidity.

The population characteristics (sex and age), geological region, and disease nature are factors to be considered in the evaluation of the threshold temperature associated with the occurrence of a disease. The effect of temperature on health could be modified by the preference of personal outdoor activities. Andrade et al. have evaluated how the ambient environment influenced the comfort of outdoor activities for population [40]. Female and the elderly prefer to have activity in a lower wind speed environment. How these potential factors influence the temperature-morbidity associations need more studies.

The intensity, duration, and timing of extreme temperature events are key factors in evaluating the health impact of extreme weather conditions [41]. Previous studies have evaluated impacts of single extreme temperature events on the morbidities of renal diseases, DM, and cardiorespiratory diseases [1]–[7]. The first extreme temperature event of the year has been linked to increased morbidity [10], [42], [43]. The present study used DLNM to simultaneously evaluate non-linear delayed temperature effects and the effects of consecutive extreme temperature events. We found a significant association between extreme cold events and ERV risks for asthma and CRF. However, extreme heat events have no significant association with any cause-specific ERV. The episodes of cause-specific ERV are much lower than that for all causes and cardiorespiratory diseases. To increase the strength of association and provide more evidences, further studies to evaluate the association between daily extreme temperatures and prolonged events and cause-specific ERV are needed.

Several reports have shown that the morbidity of renal diseases is highly associated with extreme temperatures [5], [12], [44], [45]. The primary physiological function of the kidney is to regulate the water and electrolyte balance of the body, and patients with renal impairments may not be able to adapt physiologically immediately on the exposure to an extreme high temperature. In addition, a few studies have reported that enzymatic activity throughout the human body decreases on exposure to an extreme cold temperature, which might cause renal failure and lead to death [46]–[48]. Our study found a significant association between risks of ERV for CRF and high temperature and extreme cold events, both of which can be reasonably explained by previous epidemiological or physiological studies. However, in contrast to previous findings [7], [44], [45], [49], [50], the risk of ERV for acute renal failure was not significantly associated with extreme temperature in our study (data not shown). These findings are particularly important for the health authorities in Taiwan, because the incidence and prevalence rates of end-stage renal diseases in Taiwan have ranked the first and second, respectively, in the world [51], [52].

Previous studies have reported that the increased morbidity of DM in patients exposed to extreme high temperature [9], [44], [50]. Ambient temperature may influence the blood flow and pharmacokinetics of insulin [53], which indirectly increase the risk of the disease. Even though the cumulative 4-day RR of ERV for DM was not significant in our study, the fact that the highest risk was seen on lag 0 (RR = 1.69) implies that high temperature has an immediate impact on this disease. This finding will be particularly useful in public health preparedness and intervention when DM patients expose to sudden extreme high temperature.

Only a few studies have evaluated the association between temperature and mortality from, and morbidity of, accident and injuries. The risk of accidents might be related to temperature and vary with season or climatic factors. A Japan population-based study found that occurrence of trauma, including results of motor vehicle collisions, was associated with high temperature [54], but no significant increase in accidents and injuries was observed during the 1995 Chicago heat wave [49]. In our study, we controlled for the effects of rainfall (represented by the RH) and WS and found that ERV for accidents increased as temperature increased in metropolitan Taipei, suggesting that the government should remind the public of this potential risk during hot seasons.

The risk of ERV for cerebrovascular diseases might be underestimated in this study. Fig. S1 shows the cumulative 8-day (lag 0 to lag 7) RR for ERV for cerebrovascular diseases, heart disease, or IHD at different temperatures (top row) and the RR at 14°C (middle row) or 32°C (bottom row) on different lag days for the same diseases. At 14°C, the RR for ERV for these cardiovascular diseases was the highest on lag 3 (RR = 1.12; 95% CI: 1.05, 1.20) with a cumulative 8-day RR of 1.83 (95% CI: 1.36, 2.45).

This study observed that increased exposure to PM_10_ was significantly associated with increased risk of ERV for IHD and accidents, with respective 6–day (lag 0 to lag 5) cumulative RRs of 1.28 (95% CI: 1.02, 1.60) and 1.05 (95% CI: 1.00–1.10), as the PM_10_ level increased from 31.4 µg/m^3^ (Q1) to 60.1 µg/m^3^ (Q3) (data not shown). Moreover, an increase in daily average ozone concentration from 19.0 ppb (Q1) to 31.2 ppb (Q3) was associated with the greatest 6-day cumulative risk of ERV for CRF, with a RR of 1.38 (95% CI: 1.02, 1.87) (data not shown). Increased levels of air pollutants, such as NO_2_, O_3_, and particulate matter, are significantly correlated with increased ERV for cardiorespiratory diseases [10], [55], which are regarded as important risk factors of inducing kidney morbidities [56], [57]. Moreover, Spencer-Hwang et al. reported that increased ozone levels increase the risk of coronary heart disease in kidney transplant recipients [58]. Our study did not analyze the effect of the ambient environment on patients with multiple comorbidities. The interactions among these diseases and morbidity risk from temperature and air pollution need further study.

The global temperature is in increasing trend, and extreme heat events will occur more frequent and stronger [59]. Heat wave warning systems have been widely operated in Western developed countries to reduce sudden deaths from exposing extreme heat. The findings on the associations between extreme temperatures and cause-specific morbidity [1], [9], [12]–[14], would benefit the improvement and efficiency of heat wave warning system. The present study evaluated morbidities of disease instead of all-cause or cardiorespiratory mortality in the relation with temperature extremes. Previous studies have shown that patients of kidney disorders or DM are consistently associated with high temperature [9], [14], [44], [50]. These findings suggest that the public health preventive strategy and preparedness plan for extreme temperatures should include not only preventing mortality from but also morbidity of diseases.

This study has several limitations. First, we used primary diagnostic codes to identified ERV for the temperature-cause-specific association analyses. The modification of co-morbidity could not be evaluated in this study. Second, this study didn't assess the factors that may have association with ERV, such as availability or ease of transportation, patients' willingness to travel to the hospital, modifications from other socioeconomic status, usage of air conditioning or heater, marital status, and personal income. These factors have potential influence on the temperature-cause-specific ERV association.

In summary, this study is the first to evaluate whether ERV risks of metabolic syndrome-related diseases, cardiorespiratory diseases, and accidents associated with temperature for a population residing in a subtropical climate area, controlling for potential confounders. The high daily temperature could associate with an increased risk of ERV for CRF, DM and accidents, and the low temperature could associate with an increased risk of ERV for cerebrovascular, hypertensive diseases, and asthma. A prolonged extreme low temperature increases the risk of ERV for asthma and CRF. This study suggests that events of extreme temperatures are increasing, further studies assessing threshold temperatures and physiological mechanisms associated with extreme temperatures by region and disease are in urgent needs. Public health authorities should consider the temperature effects on cause-specific diseases to improve the efficiency of warning and preparedness system.

## Supporting Information

Figure S1 **Associations between emergency room visits for cerebrovascular diseases, heart diseases, and ischemic heart diseases and daily average temperature in metropolitan Taipei from 2000 to 2009.** Top row: Cumulative 8-day (lag 0 to lag 7) RRs for cerebrovascular diseases, heart diseases, and ischemic heart diseases (IHD) estimated using DLNM and a centered temperature of 26 °. Middle and bottom rows: RR of ERV for cerebrovascular diseases, heart diseases, and IHD associated with an ambient temperature of 14°C (middle row) or 32°C (bottom row) compared to a centered temperature of 26°C on lag 0 to lag 7.
(TIFF)
