# Supplementary figures and images for: Association between Temperature and Emergency Room Visits for Cardiorespiratory Diseases, Metabolic Syndrome-Related Diseases, and Accidents in Metropolitan Taipei
Source: PLoS One. 2014 Jun 16;9(6):e99599. doi: 10.1371/journal.pone.0099599 (PMC4059706; doi:10.1371/journal.pone.0099599)

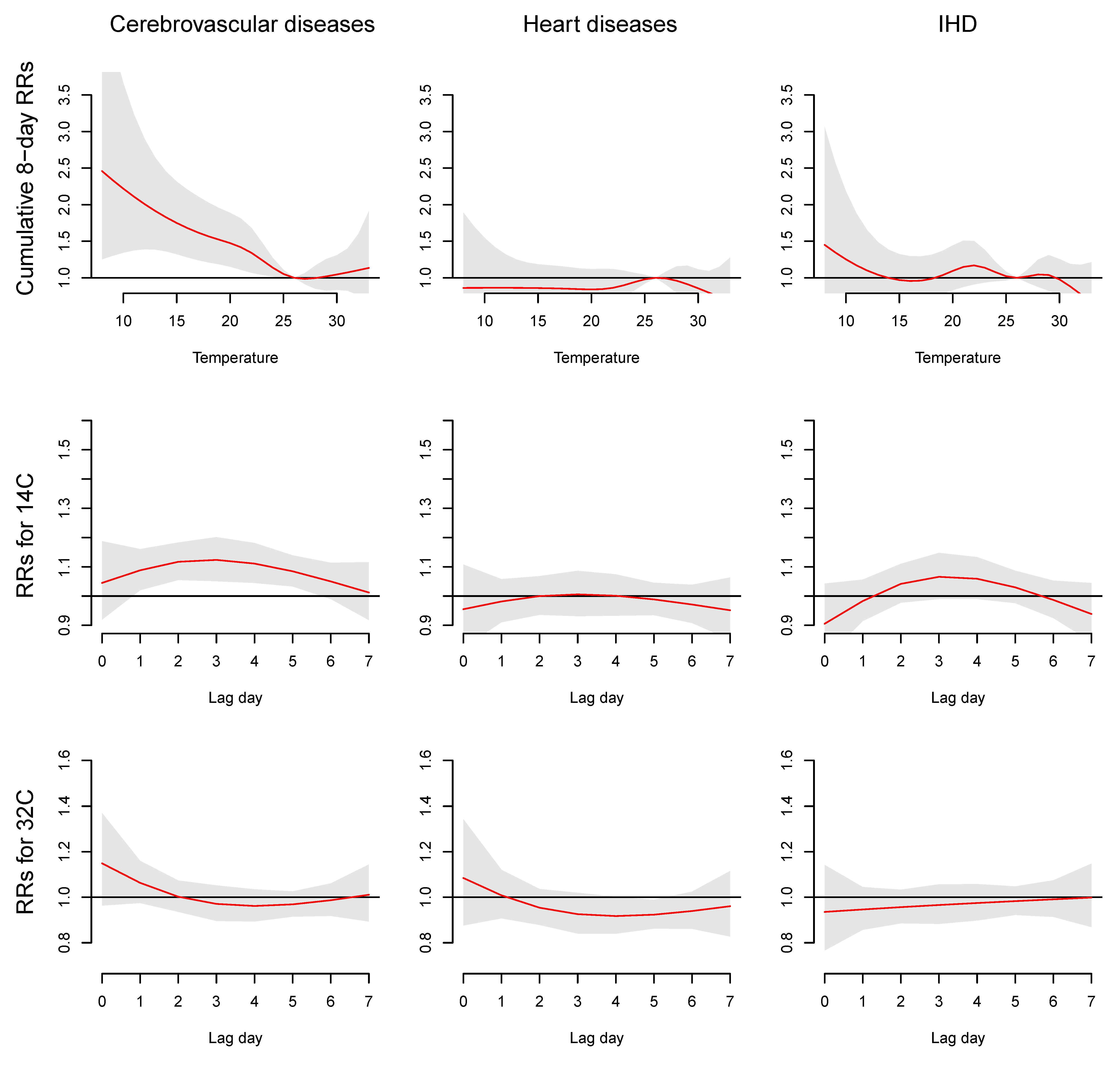

Supplement: Figure S1 — Associations between emergency room visits for cerebrovascular diseases, heart diseases, and ischemic heart diseases and daily average temperature in metropolitan Taipei from 2000 to 2009. Top row: Cumulative 8-day (lag 0 to lag 7) RRs for cerebrovascular diseases, heart diseases, and ischemic heart diseases (IHD) estimated using DLNM and a centered temperature of 26 °. Middle and bottom rows: RR of ERV for cerebrovascular diseases, heart diseases, and IHD associated with an ambient temperature of 14°C (middle row) or 32°C (bottom row) compared to a centered temperature of 26°C on lag 0 to lag 7. (TIFF) [file pone.0099599.s001.tiff]
